# Contamination of surgical mask during aerosol-producing dental treatments

**DOI:** 10.1007/s00784-020-03645-2

**Published:** 2020-10-27

**Authors:** Madline Gund, Jonas Isack, Matthias Hannig, Sigrid Thieme-Ruffing, Barbara Gärtner, Gabor Boros, Stefan Rupf

**Affiliations:** 1 Clinic of Operative Dentistry, Periodontology and Preventive Dentistry, Saarland University, Saarland University Hospital, Kirrberger Str. 100, Building 73, 66421 Homburg/Saar, Germany; 2 Institute of Medical Microbiology and Hygiene, Department of Hospital Hygiene, Saarland University, Homburg, Germany; 3 Department of Oral Surgery, German Armed Forces Central Hospital, Koblenz, Germany

**Keywords:** Surgical face mask, Infection control, Dental practice, Aerosol, Microbiology, MALDI TOF mass spectrometry

## Abstract

**Objectives:**

Surgical masks are usually contaminated during dental treatment. So far it has not been investigated whether a surgical mask itself can be a source of microbial transmission. The aim of this study was therefore to investigate the microbiological contamination of surgical masks during dental treatment and the transfer of microorganisms from the mask to the hands.

**Materials and methods:**

Five dental treatment modalities were studied: carious cavity preparation (P-caries, *n* = 10), tooth substance preparation (P-tooth, *n* = 10), trepanation and root canal treatment (P-endo, *n* = 10), supragingival ultrasonic application (US-supra, *n* = 10), and subgingival periodontal ultrasonic instrumentation (US-sub, *n* = 10). Bacterial contamination of mask and gloves worn during treatment was tested by imprinting on agar plates. Additionally, before masks were tested, their outer surface was touched with a new sterile glove. This glove was also imprinted on agar. Bacteria were identified by MALDI TOF mass spectrometry. Colony-forming units (CFU) were scored: score 0: 0 CFU, score 1: < 10^2^ CFU, score 2: > 10^2^ CFU, score 3: dense microbial growth.

**Results:**

All masks and all gloves used during treatment displayed bacterial contamination (sample scores 0/1/2/3: masks 0/46/3/1 and gloves 0/31/10/9). After touching the masks with new sterile gloves, microorganisms were recovered with the following contamination scores: P-caries: 4/6/0/0, P-tooth: 2/8/0/0: P-endo: 7/3/0/0, US-supra: 0/9/1/0, US-sub: 2/8/0/0. No statistically significant differences were detected between the treatment modalities. *Streptococci* spp. and *Staphylococci* spp. representing the oral and cutaneous flora dominated.

**Conclusions:**

Surgical masks are contaminated after aerosol-producing dental treatment procedures. Used masks have a potential to be a source of bacterial contamination of the hands.

**Clinical relevance:**

Dental staff should avoid touching the outer surface of masks with their hands to prevent transmission of pathogens. It is recommendable to change the mask after each treated patient followed by hand disinfection.

## Introduction

Dental health care professionals are exposed to numerous risk factors [1]. Mostly important are transmissions of infectious agents such as bacteria and viruses [2], but also the exposure to solvents, nanoparticles, and other substances can occur [3].

The transfer of microorganisms does not necessarily result in a risk to dental professionals. Infection and manifestation of an associated disease depend on the pathogenicity of the bacteria, the amount of them transmitted, and the current immune status of the contaminated person [1, 4]. Infectious agents can be transferred directly from the patient to the dental staff, from the dentist to the patient, and from patient to patient usually by establishing infection pathways via the staff or via (hollow) instruments, clothings, or the dental units [1, 5–7]. The highest risk for the transmission of pathogens occurs by direct blood to blood contact. However, the most common and most intensively investigated source of infection in the dental practice is aerosol. Aerosols are released in numerous dental treatment modalities [8]. These are cleaning of oral surfaces with air-water spray, preparation of carious and non-carious tooth substances with high-speed handpieces, supra- and subgingival cleaning of biofilm contaminated tooth surfaces with ultrasonic devices or powder-water spray, and endodontic and oral surgery using ultrasonic instruments [9]. These aerosols may contain microorganisms from the oral cavity or from biofilms of the dental unit, as well as blood droplets and blood-borne viruses [1, 2, 6, 10, 11]. The treatment of numerous patients per day exposes dental staff to a high frequency to this contaminative agent [12].

To protect staff and patients, numerous unspecific protective measures are applied. Dental units, surfaces, and instruments are disinfected and sterilized, aerosols are reduced by suction, and staff disinfect their hands and wear protective clothing and specific protective equipment. Also rinsing of the oral cavity using chlorhexidine or other disinfectants prior to treatment is a protective measure [4]. The protective equipment for dental staff consists of gloves, goggles, and surgical masks. For surgical interventions, hair cover and gowns are also used. Clear recommendations have been formulated for hand disinfection and the use of sterile or non-sterile gloves. These recommendations are currently formulated at the level of state-associated boards [13]. However, studies on the compliance with infection control practices in dentistry do not show very encouraging results. Hand disinfection is frequently not performed correctly before dental work or after removing gloves as defined by the guidelines [14].

In contrast to gloves, the correct use of face masks has so far not been a major issue in guidelines. There are only a few recommendations [15] and very few systematic studies on their correct use. Previous studies have focused mainly on the safety of dental health care workers against high-risk pathogens [16]. The contamination potential of surgical masks themselves was investigated in only one study. The authors have found microorganisms accumulate on the outer surface of the surgical mask when masks were used more than 2 h [17]. However, the method of usage described in this study differs fundamentally from the application in dentistry. In dentistry, (I) the surgical face mask is usually worn for shorter periods of time; (II) moreover, it is used on nearly every patient, and (III) the face mask is contaminated with microbial aerosols during almost every treatment. It has not yet been investigated if microorganisms from oral biofilms released in aerosols during dental treatment survive on the surface of face masks. Similarly, it is unclear if it is possible to transfer microorganisms from a contaminated face mask to other surfaces. The objectives of this study were therefore (I) to investigate the microbial contamination of the face mask during different aerosol-producing dental treatment modalities and (II) to analyze if touching of the contaminated face mask had a contamination potential.

## Materials and methods

### Treatment and subjects

Five typical dental treatment modalities in which aerosol release has to be expected were included in the study. Each of them comprised 10 consecutively included cases: high-speed/medium-speed preparation of a carious cavity using a rubber dam (P-caries), final high-speed preparation of caries free tooth substance for an indirect restoration without rubber dam (P-tooth), trepanation of a tooth for endodontic treatment with subsequent manual chemo-mechanical root canal preparation under rubber dam, and use of 3% sodium hypochlorite rinsing (P-endo), supragingival calculus, stain and biofilm removal with ultrasonic device (US-supra), and subgingival instrumentation with ultrasonic instruments and hand curettes (US-sub).

For the rotating instruments as well as for the ultrasonic application, dental unit water was used for cooling. Evacuation was established by means of conventional dental suction (CDS) using a cannula of 3.3 mm in diameter (suction flow 1.1 l/s) and high-volume evacuation (HVE, tube of 8.0 mm in diameter, suction flow 6.0 l/s). The CDS was placed lingually from the lower central incisors, even if rubber dam was used. The HVE was held by an assistant.

Dental practitioners (*n* = 14) wore sterile gloves (Gammex Latex, Ansell, Brussels, Belgium), surgical face masks (tie-band medical face mask type II, Mölnlycke Health Care, Düsseldorf, Germany), and protection eyewear (Safeview eyewear, Halyard, Neunkirchen, Germany). Hygienic hand disinfection was executed before the protective equipment was applied. Direct contact with the skin, oral mucosa, and teeth of the patients was avoided. All instruments were sterile. The dental unit and the surrounding surfaces were disinfected by wiping (Celtex Wipes, Lotfex, Bremen, Germany). The room temperature was 20‑22 °C with 40‑60% relative humidity.

Only patients without known infectious diseases were included in the study. No individual patient or practitioners’ data were recorded. All samples were anonymized. Verbal informed consent was obtained from all participants. Ethical approval for the study was obtained from the Ethics Committee of the Saarland Medical Association (Vote No. 181/19).

### Sampling

**Fig. 1:**
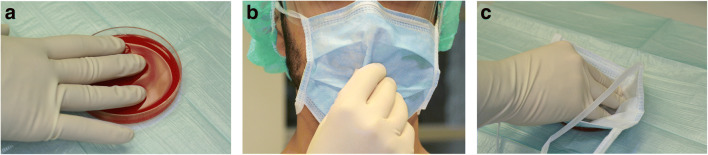
Microbiological testing of gloves and face mask worn during treatment. **a** Direct contact sample of gloves worn during treatment, pressing three digits for 5 s on agar plate. **b** Evaluation of a contamination pathway by touching the mask for 5 s with a new sterile glove and imprinting this glove according to **a** and **c**. Imprint of the mask on agar to analyze contamination with microbials containing aerosols

Microbiological sampling was conducted 30 min after affixing of the surgical mask and immediate starting of aerosol-releasing dental work. Three samples were collected from each treatment session. (I) Microbial aerosol contamination was examined on the face mask. (II) Direct contamination was tested on gloves worn during treatment. (III) Finally, an indirect path of contamination was evaluated by touching the used face mask for 5 s with a new sterile glove. The order of microbiological sample collection was as follows: first, glove worn during treatment (Fig. 1a), second, new sterile glove after touching the face mask (Fig. 1b), and third, face mask worn during treatment (Fig. 1c).

Unused sterile face masks and new sterile gloves (*n* = 5 each) served as negative controls. Additionally, sterile face masks (*n* = 15) worn by three dentists for 30 min were used to control the contamination from the dentist’s oral cavity. To perform these tests, the subjects were sitting alone, breathing quietly, in a ventilated clean room without speaking or coughing.

All face masks and gloves were directly imprinted for 5 s on brain heart infusion (BHI) agar plates (Karl Roth, Karlsruhe, Germany, diameter: 90 mm, Fig. 1 a, b). Gloves were tested directly by an imprint on the agar plate without removal of the gloves. Face masks were removed by an assisting person wearing sterile gloves without touching the surface.

### Culture and identification of bacteria

All agar plates were immediately incubated at 35 °C ± 2 °C with 5% CO_2_ for 48 h. Plates were read after 24 h and 48 h. After 48 h, all phenotypically different colonies were classified using matrix-assisted laser desorption/ionization time-of-flight mass spectromy MALDI TOF MS (Microflex LT/SH, Bruker Daltonik, Bremen, Germany), FlexControl, and MALDI Biotyper Compass software packages (Bruker Daltonik). Colonies were picked and transferred to a stainless steel target (96-spot target, Bruker Daltonik) using a toothpick and overlayed with 2 μl of matrix (alpha-cyano-4-hydroxycinnamic acid, 20 mg/ml in 0.1% trifluoroacetic acid (TFA)/acetonitrile 1:2). Samples were allowed to crystallize, washed twice with 0.1% TFA, and re-crystallized in 0.1% TFA/acetonitrile 1:2. Measurements were carried out in linear positive mode (delay 400 ns, voltage 20 kV, mass range 2–20 kDa). The spectra were externally calibrated with the standard calibrant mixture, Protein Calibration Standard I, supplied by Bruker Daltonik. Two hundred forty laser shots were applied *per* spot. The measurements were continued until the bacterium was clearly identified. If a spectrum could not be assigned to a known species, it was noted as “unidentified”.

The colony numbers on the agar plates were classified with a scoring system. No bacterial growth was rated as score 0, 1–100 scattered and countable colonies (colony-forming units: CFU) as score 1, > 100 countable CFU as score 2, and agar plates displaying areas with dense microbial growth with uncountable colonies in a bacterial lawn as score 3.

### Data analysis

The qualitative and quantitative results of the bacterial contamination tests were presented descriptively. Colony scores of the samples from gloves and masks were statistically compared with the Mann-Whitney *U* test (*p* < 0.05). The Kruskal-Wallis test was used to compare treatment modalities (*p* < 0.05).

## Results

### Contamination rates and scores

All unused sterile face masks and gloves displayed no bacterial growth. In addition, the masks worn to control the infection from the dentist’s oral cavity showed no contamination of their outer surface.

**Fig. 2:**
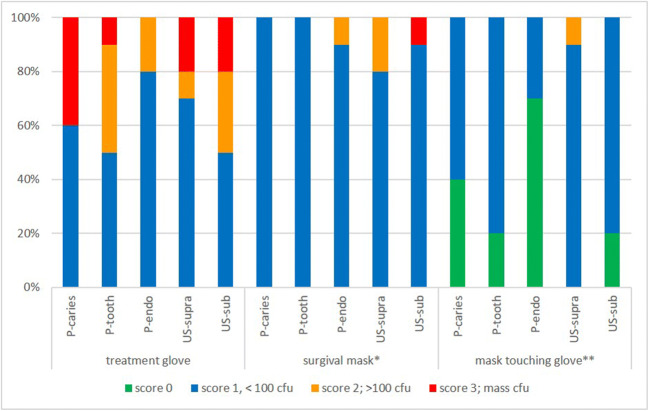
Summary of the detection frequency and scoring of microbes on agar plates after sampling of gloves worn during treatment, surgical masks, and Mask-touching gloves. The columns each represent the 10 microbiological samples of the 5 treatment modalities carious cavity preparation (P-caries), tooth substance preparation (P-tooth), trepanation and root canal treatment (P-endo), supragingival ultrasonic application (US-supra), subgingival periodontal ultrasonic instrumentation (US-sub). The columns are arranged according to the CFU scoring (score 0: no bacterial growth; score 1: 1–100 scattered CFU; score 2: > 100 CFU; score 3: dense microbial growth). No statistically significant differences were found between the different treatment modalities (treatment glove: *p* = 0.7, surgical mask: *p* = 0.9, mask-touching glove: *p* = 0.3). The contamination of surgical masks was significantly lower than of treatment gloves (*, *p* = 0.008). Mask-touching gloves displayed significantly lower contamination than surgical masks (**, *p* = 0.004)

Bacterial contamination was detected on all 50 face masks worn during the five treatment modalities. A low bacterial growth with less than 100 CFU (score 1) was found on 46 face masks, more than 100 CFU (score 2) on three masks, and a dense growth with uncountable colonies (score 3) for one mask. All 50 samples of gloves worn during treatment similarly showed bacterial contamination. Score 1 was recorded for 31 gloves, score 2 for 10 gloves, and score 3 for 9 gloves. The fresh sterile gloves, with which the face masks were touched after treatment, displayed no contamination (score 0) in 15 samples, score 1 in 34 samples, and score 3 in one sample. The results of the CFU scoring for the 5 treatment modalities are presented in detail in Fig. 2.

The comparison of the contamination scores revealed significantly higher numbers of colonies in gloves used during treatment compared to surgical masks (*p* = 0.008), between these gloves and the mask-touching gloves (*p* < 0.00001) as well as between masks and the gloves touching the masks (*p* = 0.004). Differences between treatment modalities were not statistically significant (treatment gloves: *p* = 0.7, surgical masks: *p* = 0.9, mask-touching gloves: *p* = 0.3).

### Identified bacteria

The microorganisms identified in this study are presented in Table 1. The genus *Staphylococcus* showed the highest prevalence. Of this genus, the species *Staphylococcus epidermidis*, *Staphylococcus capitis*, *Staphylococcus hominis*, *Staphylococcus aureus*, *Staphylococcus saprophyticus*, *Staphylococcus warneri*, *Staphylococcus caprae*, and *Staphylococcus pettenkoferi* were detected in decreasing frequency. The genus *Streptococcus* (*Streptococcus oralis*, *Streptococcus mitis*, *Streptococcus anginosus*, *Streptococcus sanguinis*, *Streptococcus parasanguinis*, *Streptococcus salivarius*, *Streptococcus gordonii*, *Streptococcus infantis*), *Bacillus* (*Bacillus cereus*, *Bacillus circulans*, *Bacillus subtilis*), *Micrococcus* (*Micrococcus luteus*), *Rothia* (*Rothia dentocariosa*, *Rothia aeria*), *Neisseria* (*Neisseria macacae*, *Neisseria perflava*, *Neisseria subflava*, *Neisseria oralis*), *Penicillium* (*Penicillium chrysogenum*), *Actinomyces* (*Actinomyces oris*), and *Pseudomonas* (*Pseudomonas monteilii*, *Pseudomonas stutzeri*) were also detected with several species. Further species (*Acinetobacter pittii*, *Aerococcus viridans*, *Aspergillus versicolor*, *Dermabacter hominis*, *Enterococcus faecalis*, *Haemophilus parainfluenzae*, *Rhodococcus erythropolis*, *Sphingomonas* sp., *Streptomyces* sp.) were found sporadically. Masks and gloves which were in contact with the masks showed comparable microbial patterns, with a smaller frequency of the species. Also here *Staphylococcus* spp., *Streptococcus* spp., and *Bacillus* spp. dominated. Importantly, in three samples, two face masks and one treatment glove, *S. aureus* was detected. These strains were methicillin sensitive as shown by susceptibility testing. Concerning the bacterial species, no systematic differences were apparent between the treatment modalities.

**Table 1:**
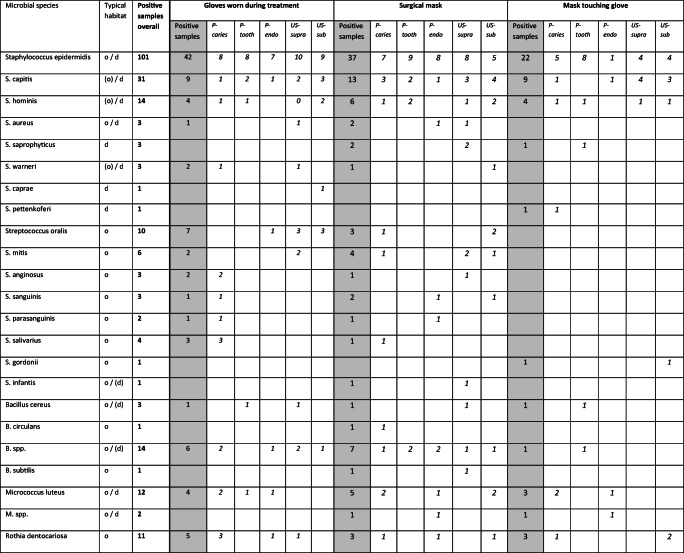

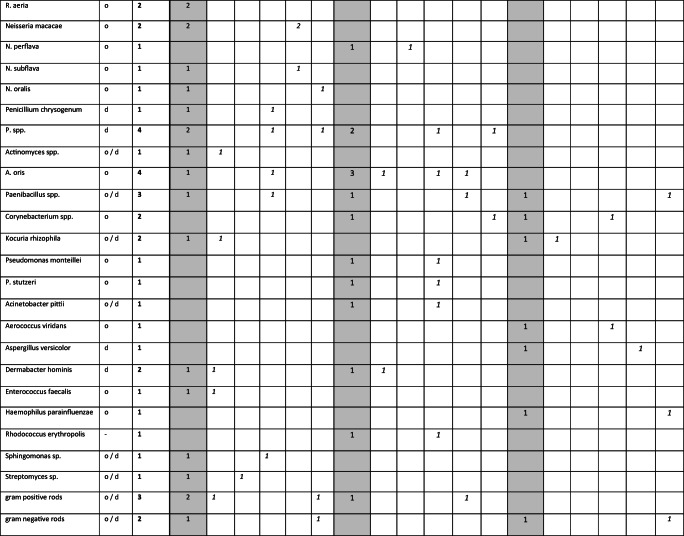
Species of microorganisms classified within this study and frequency of their detection from gloves and masks. The typical habitat in human is indicated (oral: o, dermal: d, low frequency: in branches). The total number of all positive samples (max. *n* = 150, **bold**), as well as from gloves worn during treatment, surgical masks, and glove-touching masks (max. *n* = 50 each, gray), is indicated. Microbial species detected on gloves and masks are presented according to the treatment modalities (max. *n* = 10 each, *italics*) carious cavity preparation (P-caries), tooth substance preparation (P-tooth), trepanation and root canal treatment (P-endo), supragingival ultrasonic application (US-supra), and subgingival periodontal ultrasonic instrumentation (US-sub). Colonies only identified on upper taxonomic levels are indicated as spp. or as gram positive or gram negative rods

## Discussion

Two important findings were obtained through this study. First, we proved that microorganisms survive on surgical masks as used during dental work for a timeframe of at least about 30 min. Secondly, we demonstrated that viable microorganisms can be recovered by the hands from the contaminated masks and transferred to a susceptible surface. The study has thus proven a contamination pathway that has not been described so far. From the results of this study, it can be assumed that there is a risk of infection from a contaminated surgical mask via the hands to any other surfaces or to humans in dental facilities.

We showed clearly in our study that the hands could be contaminated by touching a contaminated face mask. This might result in a contamination of the dentist’s hand with microorganisms from the patient treated before with a relevant risk for the next patient or the therapeutist itself. As a consequence, face masks must be discarded after each patient. In addition, the hands must be disinfected after touching of the face mask, e.g., if the mask is removed after an aerosol-producing therapeutical procedure to talk to the patient and replaced for the next therapeutical step.

Within this study, aerosol-releasing treatment techniques were applied which are used typically several times in daily dentistry. The usual measure to prevent the dissemination of aerosols, the high-volume evacuation, was also applied. The evacuation proved not to be able to achieve a complete prevention of contamination of the outer surface of the surgical masks. This result confirms former studies on the exposure of the dentist to fine particles or microorganisms despite using the high-volume evacuation [18]. This underlines the importance of the mask for the protection of dental staff. Remarkably, the different treatment modalities showed no significant differences in microbial contamination of the mask and the transfer of microorganisms from the mask to the sterile gloves. The surgical mask seems to provide excellent conditions for the survival of oral or dermal bacteria. This could be caused by the humidity of the exhaled air of the mask wearer. Moreover, the temperature of the exhaled air, which is almost body temperature, is perfect for the replication of many bacteria. In a previous study [17], it was found that when wearing surgical masks for more than 2 h, an increasing number of microorganisms from the environment or from the oral cavity and respiratory system of the mask wearer accumulate. In the present study, sterile masks were worn for 30 min. No viable microorganisms could be detected in these control experiments. Although this result is in line with another study [17], it cannot be ruled out that, in addition to the patient’s own microorganisms, microorganisms from the dentist’s oral cavity may also reach the outer layer of the mask through the face mask. However, this limitation is not relevant for the conclusions of this study.

The majority of the microorganisms found in our study were typical bacteria of the oral or dermal microbiome. The species with the highest prevalence in this study was *S. epidermidis*. It was detected on two-thirds of the studied surgical masks and gloves (Table 1). One-fifth of masks and gloves showed contamination with other *Staphylococci* spp. The species *M. luteus*, *R. dentocariosa*, *S. oralis*, and *Bacillus* spp. were each detected on more than ten masks and gloves.

Although the contamination scores for the masks and the sterile gloves that touched the masks were significantly lower than that for gloves worn during dental work, a similar microbiological spectrum was identified.

The sampling technique and microbiological methodology used in the study had strengths and limitations. One strength of the cultivation of microorganisms on BHI agar is the exclusive detection of viable microorganisms. Non-viable bacteria are not able to cause infections. The selected agar, like any other microbiological substrate, has selective properties but is usually employed to detect the vast majority of fast-growing species. Obligate anaerobic microorganisms are not recorded, nor are various slowly growing bacteria. For MALDI TOF MS diagnostics, only colonies with different phenotypes were selected. This might led to an underestimation of the microbial spectrum. In cases of dense microbial growth, several areas were analyzed. However, competitive growth may also have reduced the detected microbial spectrum. The technique of microbiological sample collection may also underestimate contamination and microbial diversity on masks and gloves. The surgical mask is made of air-permeable material. The contact sample on agar probably only detects the microbes adhering to the surface. The gloves on the agar plates with an inner diameter of 90 mm could also not be completely brought into contact with the culture medium. In summary, the total numbers of viable microorganisms on both the mask and the gloves must be expected to be higher in real-life settings than in our study.

Some specific bacteria that were detected on masks and transmitted via the hands to agar plates will be discussed in the following. Each of the coagulase-negative staphylococci such as *S. epidermidis* or even *S. aureus* is potentially multi-resistant bacterial species. The prevalence of *S. aureus* was low in this study and lower than found in a previous study [19]. This species was only detected in three samples. Reasons for this could be that only patients who did not report general diseases were enrolled in this study and dental staff were informed and were highly compliant with the hygienic standards in our clinic. Reports are available in the literature showing a higher prevalence of *S. aureus* in dental students than in control groups [20, 21], but other studies revealed lower carriage rates [16]. Nevertheless, this species naturally represents a risk pathogen and in this study, a possible transmission path for *S. aureus* was demonstrated.

The most frequently isolated microorganism in this study was *S. epidermidis*. It was detected in 22 of the 50 microbiological samples obtained from gloves touching the surgical mask. This high detection frequency is in line with the results of other studies [22]. *Staphylococcus epidermidis* is the most common member of coagulase-negative staphylococci on human epithelial surfaces and it has to be regarded as an important nosocomial pathogen [23].

The other detected microorganisms such as *S. capitis*, *S. oralis*, *M. luteus*, or *R. dentocariosa* and all others are oral or dermal bacteria of the commensal flora. All these microorganisms are not pathogenic to healthy individuals, but may be hazardous to immunosuppressed or immunocompromised patients. Since in individual cases the health status of the patient and the risk factors for a facultative pathogenic species to become pathogenic are not always clear, it would be reasonable to consistently implement compliance with regulations and recommendations for the prevention of nosocomial infections [14]. For an infection and a clinical manifestation of a disease in a dentist or dental staff, both frequency of exposure and the virulence of the pathogen are of importance [1]. As a consequence, constant preventive behavior is of high importance since it is not possible to assess in dental practice whether a patient is colonized with pathogenic or facultative pathogenic microorganisms that can be transferred in the respective dose to a susceptible dental health care professional.

In this study, the contamination of face masks by viruses was not investigated. This represents an additional risk for dental staff and patients. However, in the context of the COVID-19 pandemic, general hygiene measures and those aimed at preventing the transmission of microorganisms should not be compromised.

## Conclusion

The results of the study confirm that surgical masks are contaminated after aerosol-producing dental treatment procedures. As a consequence, masks are a potential source of contamination of gloves or the hands when touching a used surgical mask. The mask, which is useful for personal protection, should be discarded after each patient contact. Touching the outer surface of the mask should be avoided at any time. After touching or removing the mask, the hands must be disinfected.
